# The type and extent of travel for professional footballers undertaking national team duties for a national football federation

**DOI:** 10.5114/biolsport.2023.119288

**Published:** 2022-10-06

**Authors:** Ewan Clements, Fabian Ehrmann, Andrew Clark, Mark Jones, Donna Lu, Rob Duffield

**Affiliations:** 1 School of Sport, Exercise and Rehabilitation, Faculty of Health, University of Technology Sydney, Australia; 2 Football Australia, Sydney, Australia

**Keywords:** Soccer, Jet lag, Travel fatigue, National team transition, Performance

## Abstract

Elite football (soccer) involves club, continental and international fixtures, requiring players to undertake extensive travel [1]. For a national football federation, this includes the transport of players between club and camp/tournament commitments, which is often a point of contention between respective organisations [2]. Partly this contention results from the effects of travel, whereby jet lag and travel fatigue can negatively affect physical performance [3–5] and athlete wellbeing [6, 7]. Given the scarcity of data on elite players following travel, an initial step for any national football federation is to understand the volume and nature of travel undertaken by national team players. Such insight may better identify the schedule, timelines and needs of athletes’ post travel. Better awareness of these travel needs can help maximise availability for training and minimise the impact of travel related stresses on performance or wellbeing. However, the regularity and volume of travel to national football team commitments has not previously been described. Further, travel demands are likely to vary significantly based on the location of the athlete and the national team camp. For countries outside of Europe, such as Australia, the travel demands and ensuing effects on player preparation can be substantial for both arrival into national team and on return to clubs [7]. Hence, detailed information regarding the type, frequency, and extent of travel for national team duties is important to aid in planning optimal travel schedules and interventions to assist players for international or club duty.

## INTRODUCTION

In the absence of player data in the research literature, specific detail related to national team travel demands is needed, as the influence of jet lag and travel fatigue will differ based on a number of different factors related to the journey. Time zone shifts of > 3 h are likely to induce symptoms of jet lag, though athletic performance reductions exist with greater time zone differences [8, 9]. For example, reductions in intermittent and maximal sprint performance [4] and jump performance [3, 4] are observed after time zone shifts of > 8 h. Similarly, long-haul travel of > 22 h can reduce sleep duration [5, 6, 10, 11], which may explain elevated fatigue, [6, 10] and reduced intermittent sprint performance [6] and lower body power [5] following arrival. In contrast, northbound travel of 10 h where athletes did not travel overnight had negligible effects on sleep and wellness [12]. The lack of effect from this flight may be attributed to the northward direction of travel and thus lack of significant time zone change, while it is also possible that the timing of the flight relative to the sleep period may be more critical than the duration of travel. Thus, flights of roughly > 10 h with time zone changes of > 3 h should be of concern given the likelihood of inducing jet lag symptoms or interrupting normal sleep cycles. Understanding the frequency and extent of potentially problematic travel may assist national football federations in planning training schedules and recovery interventions following arrival.

Currently, only two studies report travel in national football teams, with trips of 15 h across 4 time zones [13] and 19 h across 11 time zones [7]. Separately, the travel schedules of Australian club sides competing in Asian continental competitions report travel durations of 10 h [12] and 25.6 h [11]. However, these reports do not describe the full range of travel demands likely to be experienced or allow planning for the diversity of demands for national team players. Furthermore, it is possible that the travel demands for players contracted to clubs outside of their home continent are greater than players within the national domestic competition. For a national football federation based outside of Europe, such as Australia, a large number of national team players are contracted to European and Asian clubs. A greater understanding of the travel demands of national team players based on club location can inform tailored travel schedules and interventions based on specific needs. Accordingly, the aim of this study is to describe the nature and extent of travel performed by Australian national team football players for international duties over a two-year period. In addition, this study compared the travel demands for national duties between players based in Australian (domestic), Asian and European club locations.

## MATERIALS AND METHODS

### Participants

Participants were 58 male senior Australian national football (soccer) team representatives who had undertaken travel to train or compete for the national team between March 2018 and November 2019. Through contractual agreements, participants provided consent to Football Australia for the use of their anonymous data for research purposes, and human ethics approval was provided by the institutional Human Ethics Committee (ETH20–5080).

### Data Collection

Details of all travel schedules undertaken as a part of Australian national team duties between March 2018 and November 2019 were provided by Football Australia. This included the details of 569 different trips (including multiple flights per trip). Of note, during this period the Australian team competed in the 2018 FIFA World Cup Finals in Russia, the 2019 AFC Asian Cup Finals in the United Arab Emirates, and the Round 2 qualifying process for the 2022 World Cup. Participant data was anonymised prior to being provided with player names replaced by numerical codes.

All flights were provided based on booked travel schedules, which were then independently verified to obtain the actual arrival and departure times through an online flight database (Flightera.net). For each listed trip, the following data was extracted, i) total flight time ii) total travel time iii) time zone change iv) number of overnights per trip v) departure time vi) arrival time vii) number of trips per player and viii) generic direction i.e. East/West. In defining these variables, arrival and departure time relate to the specific time the aircraft took off and landed, as reported via the online flight database. Total flight time was measured as the duration of all flights included in the journey from departure to arrival location. The total travel time was the difference between departure time and arrival time, and included both flight time and stop-over time, however, did not include any additional travel requirements outside air-travel. The time zone change was calculated based on the difference between the arrival and departure time zone on the day in which the player arrived and coded for direction as East, West, or No change. A trip was considered to have occurred overnight if the arrival time was later than midnight of the day of departure. The geographical continent in which a player competed at club level at the time of travel was provided by Football Australia and was used for comparisons between the travel demands of players in Australia, Europe and Asia. All flight measures for each player were collated in a Microsoft Excel spreadsheet and time-based measures were converted into a decimal of hours (i.e. 12 h 30 min was equal to 12.5 h). Time-based variables are reported in 3 h groupings to better report the range and frequency of travel demands. For comparisons between club locations, all flights were labelled as being either outbound (travelling to national team duty, n = 244) or return (returning to club from national team, n = 244). Transition trips between national team commitments or to a location other than the players club were excluded from location-based comparisons (n = 71).

### Statistical Analysis

Descriptive data for mean, standard deviation, median, minimum, and maximum values for all flight variables are reported. Shapiro-Wilk normality tests demonstrated that the data was not normally distributed, and comparisons between club location groups for all travel variables were assessed using non-parametric Kruskal-Wallis tests from the “stats” package in R [14]with significance set at *p* < 0.05. Where a significant difference was observed between the groups, pairwise comparisons were made using Dunn Tests [15] with Holm corrected *p* values. All statistical tests were performed in the R statistical software [14].

## RESULTS

**FIG. 1:**
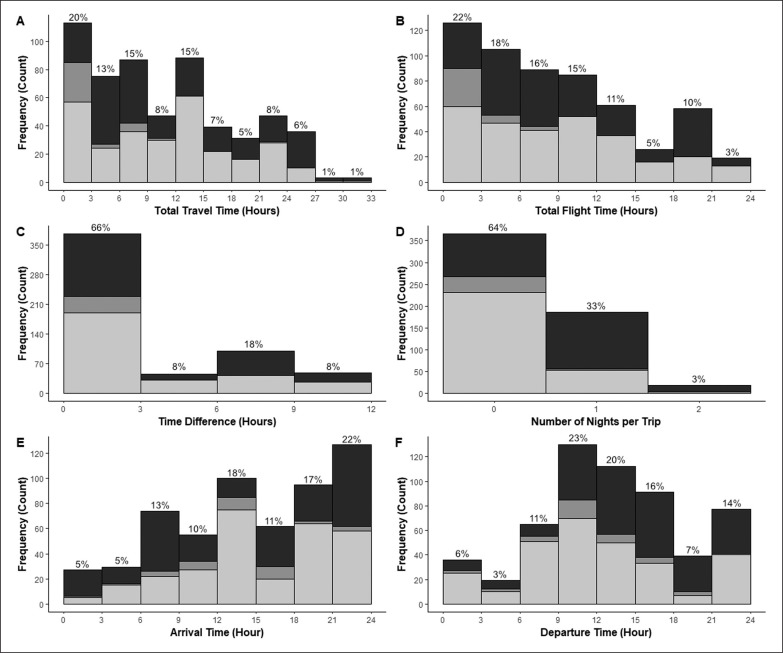
Distribution of A) Total Travel Time B) Total Flight Time C) Time Difference D) Flights per Trip E) Arrival Time F) Departure Time for national team footballers during a twoyear period (n=569) for Eastward (Dark), Westward (Light) time zone shifts, and No time change (Grey). Percentages above bars represent the proportion of each bar relative to all trips.

A majority of trips involved time zone differences of ≤ 3 h (66%), though 34% of flights involved differences of > 3 h and 17% of flights involved large time differences of ≥ 8 h (Figure 1). The direction of time shifts included 50% westward travel, 43% eastward and 7% without any change in time zone. Travel times of ≥ 10 h occurred in 51% of trips, while 8% involved ≥ 24 h travel time. For flight durations, 41% of trips involved ≥ 10 h flight time, while 7% involved ≥ 20 h flight time. Most flights (64%) did not include overnight travel, while 33% involved one night and 3% involved two nights. The most common arrival time was in the evening between 18:00 and 24:00 (39%), with early morning arrivals between 24:00 and 09:00 occurring for 23% of flights and 39% of flights arrived during the day (09:00 to 18:00). Players most often departed during the day between 09:00 and 18:00 (59%), while 21% of trips departed in the evening (18:00–24:00) and 20% of trips departed in the early morning (24:00–09:00).

A significant effect of player location on time zone change was observed for both outbound (H = 10.18, *p* = 0.006; Table 1) and return (H = 7.505, *p* = 0.023; Table 2) travel. Asian-based players crossed significantly fewer time zones than Australian- (*p = 0.042)* and European-based players during outbound travel (*p = 0.004)*, and Australian-based (*p* = 0.018) players during return travel.

**TABLE 1:** Travel demands of Australian professional footballers based on club geographical location travelling to a national team commitment over a two-year period (n = 244)

Measure	Player Location	Mean (± SD)	Median	Min.	Max.
Trips per Player (N)	All Players	4.1±2.4	3.5	1.0	10.0
	Australia	3.2±1.9	3.0	1.0	7.0
	Europe	4.6±2.6	4.5	1.0	10.0
	Asia	3.2±1.6	3.0	1.0	7.0

Flight Duration (Hours)	All Players	9.2±6.4	6.7	0.8	23.0
	Australia	12.1±6.5^*#^	12.8	0.9	20.8
	Europe	8.9±6.4	6.4	0.8	23.0
	Asia	7.9±5.4	7.5	1.2	17.8

Travel Duration (Hours)	All Players	11.1±8.1	7.7	0.8	31.0
	Australia	14.3±8.0	14.1	0.9	27.6
	Europe	10.7±8.2	6.9	0.8	31.0
	Asia	9.5±6.4	10.0	1.2	21.5

Time Difference (Hours)	All Players	3.8±3.3	2.0	0.0	10.0
	Australia	4.3±3.5^#^	5.0	0.0	10.0
	Europe	3.9±3.3^#^	2.0	0.0	10.0
	Asia	2.6±3.0	1.0	0.0	9.0

Eastward Trips per Player (N)	All Players	2.8±2.5	2.0	0.0	8.0
	Australia	0.2±0.4^*^	0.0	0.0	1.0
	Europe	4.1±2.3^#^	4.0	0.0	8.0
	Asia	1.3±0.9	1.0	0.0	3.0

Westward Trips per Player (N)	All Players	1.0±1.5	0.0	0.0	7.0
	Australia	2.8±1.7^*^	3.0	1.0	7.0
	Europe	0.3±0.7	0.0	0.0	2.0
	Asia	1.2±1.3	1.0	0.0	3.0

Overnight Trips per Player (N)	All Players	2.3±1.8	2.0	0.0	7.0
	Australia	1.5±1.4	1.0	0.0	5.0
	Europe	2.7±2.0	2.5	0.0	7.0
	Asia	1.3±1.2	1.0	0.0	3.0

Arrival Time (HH:mm)	All Players	13:54±07:18	14:59	00:00	23:54
	Australia	12:42±05:42	11:48	00:18	22:18
	Europe	14:24±07:48	16:54	00:00	23:54
	Asia	11:48±05:36	11:12	00:54	21:18

Departure Time (HH:mm)	All Players	14:36±05:36	14:42	00:12	24:00
	Australia	13:06±07:42	10:42	00:12	23:42
	Europe	15:00±04:42	15:00	02:06	23:00
	Asia	13:48±06:42	11:54	00:36	24:00

* Significantly different to Europe (p < 0.05);

# Significantly different to Asia (p < 0.05)

**TABLE 2:** Travel demands of Australian professional footballers based on club geographical location returning to club teams following a national team commitment over a two-year period (n = 244)

Measure	Player Location	Mean (± SD)	Median	Min.	Max.
Trips per Player (N)	All Players	4.1±2.4	4.0	1.0	10.0
	Australia	3.3±1.9	4.0	1.0	8.0
	Europe	4.7±2.7	4.0	1.0	10.0
	Asia	3.1±1.4	3.0	1.0	6.0

Flight Duration (Hours)	All Players	9.6±5.8	8.5	0.6	21.8
	Australia	12.2±5.3^*#^	12.9	1.2	19.3
	Europe	9.3±5.8	7.7	0.6	21.8
	Asia	7.2±5.3	4.6	0.9	18.3

Travel Duration (Hours)	All Players	12.2±7.3	11.1	0.6	30.7
	Australia	15.9±7.0^*#^	16.4	1.2	29.2
	Europe	11.7±7.1	10.3	0.6	30.7
	Asia	9.3±7.0	8.0	0.9	23.9

Time Difference (Hours)	All Players	3.7±3.3	2.0	0.0	11.0
	Australia	4.8±3.5^#^	6.5	0.0	10.0
	Europe	3.6±3.3	2.0	0.0	11.0
	Asia	2.8±3.2	1.0	0.0	9.0

Eastward Trips per Player (N)	All Players	1.2±1.4	1.0	0.0	6.0
	Australia	2.8±1.6^*^	3.0	1.0	6.0
	Europe	0.6±0.9	0.0	0.0	3.0
	Asia	1.3±0.9	1.0	0.0	3.0

Westward Trips per Player (N)	All Players	2.5±2.4	1.5	0.0	8.0
	Australia	0.2±0.4^*^	0.0	0.0	1.0
	Europe	3.8±2.2^#^	4.0	0.0	8.0
	Asia	0.9±0.6	1.0	0.0	2.0

Overnight Trips per Player (N)	All Players	1.3±1.4	1.0	0.0	5.0
	Australia	2.8±1.8^*#^	3.0	0.0	5.0
	Europe	0.9±1.0	1.0	0.0	3.0
	Asia	1.0±1.2	1.0	0.0	3.0

Arrival Time (HH:mm)	All Players	15:00±05.48	17:00	0.1	23.9
	Australia	13:06±06.24^*^	18:00	04:54	22:36
	Europe	15:36±05.30	11:00	01:00	23:54
	Asia	14:48±05:54	17:12	00:06	23:24

Departure Time (HH:mm)	All Players	11:48±06:00	10:54	00:30	23:42
	Australia	13:12±06:00	10:06	01:36	23:18
	Europe	11:06±06:00^#^	14:36	00:30	23:12
	Asia	13:48±05:00	13:48	03:24	23:42

* Significantly different to Europe (p < 0.05).

# Significantly different to Asia (p < 0.05)

Significant differences for total travel time existed for both outbound (Table 1) and return (Table 2) travel (Outbound: H = 6.159, *p* = 0.046; Return: H = 16.754, *p* < 0.001) and for total flight time (Outbound: H = 7.580, *p* = 0.023 Return: H = 16.221, *p* < 0.001). Travel time was significantly greater in Australian- compared to European-based players for return travel (*p* = 0.001) and neared significance for greater outbound travel duration (p = 0.073). Australian-based players had significantly greater return travel duration (*p* = 0.001) and neared significance for greater outbound travel duration (*p* = 0.064). Total flight time for both outbound and return groups was significantly greater for Australian-based players (European Outbound: *p* = 0.030 Return: *p* = 0.003; (Asian Outbound: *p* = 0.043 Return: *p* = 0.001).

The number of overnight trips per player was significantly different between groups for both outbound (H = 6.066, *p* = 0.048) and return (H = 11.850, p = 0.003). With Bonferroni correction, no pairwise comparisons reached significance for outbound travel, while Australian-based players travelled overnight more frequently than both European- (p = 0.002) and Asian- (*p* = 0.046) based players during return travel. Significant differences existed in arrival time for both outbound (H = 6.597, *p* = 0.037) and return travel (H = 6.567, *p* = 0.038); however, with Bonferroni correction, no pairwise comparisons for outbound travel reached significance (*p* > 0.05). For return travel, Australian-based players arrived significantly earlier in the day than European-based players (*p* = 0.035). Significant differences existed in departure time for return (H = 9.556, *p* = 0.008), but not outbound travel (H = 2.050, *p* = 0.359). Post-hoc analysis showed significantly earlier departure times for European compared to Asian-based players (p = 0.049) for return trips.

No significant differences existed for the total number of trips per player for outbound (H = 3.967, *p* = 0.138) or return (H = 3.694, *p* = 0.158) travel. Significant differences existed in the number of trips in both eastward (Outbound: H = 31.282, *p* < 0.001 Return: H = 20.497, *p* < 0.001) and westward (Outbound: H = 28.667, *p* < 0.001 Return: H = 31.468, *p* < 0.001) directions. European-based players completed significantly more outbound eastward trips than both Australian (p < 0.001) and Asian-based players (p = 0.016) and significantly less westward outbound trips than Australian- (p < 0.001) and Asian-based (p = 0.074) players. For return trips, European-based players completed significantly more westward trips than both Australian (*p* < 0.001) and Asian-based (*p* = 0.009) players, and significantly fewer eastward trips than Australian-based players (p < 0.001).

## DISCUSSION

This study describes the type and extent of travel demands for Australian national team duties and compares travel demands based on a player’s club location. A large number of trips by national team players are unlikely to affect performance and wellbeing (66% ≤ 3 h time difference, 64% not overnight, 49% < 10 h travel time). Despite this, a number of flights exceed 3 h of time difference (34%), occur overnight (36%) or are prolonged in duration (51%, > 10 h) and therefore potentially pose concerns for performance or recovery. Being aware of the frequency of extensive travel demands may in turn allow national team staff to better prepare for the arrival of players and guide preventative measures before and after travel. Furthermore, Australian-based players generally had greater travel demands than Asian or European-based players. Therefore, travel strategies should consider location-specific demands of players; with those travelling into the national team from Europe or returning to Australian-based clubs needing greater attention for circadian adaption and promotion of sleep assistance strategies.

This study shows a number of trips resulting in time zone differences of > 3 h (34%), which have been previously observed to induce jet lag symptoms in athletes [13, 16–18], though symptoms are expected to be more detrimental with greater time zone differences [19–21]. Although no performance measures were recorded in this study, 17% of trips, exceeded 8 h of time zone difference, with such time zone changes previously being shown to cause reductions in intermittent and maximal sprint as well as jump performance [3, 4]. This study highlights that many national team trips for this federation have the potential to induce detrimental jet lag symptoms and thus practitioners should consider interventions that can hasten the rate at which an athlete adapts to time zone changes. Further, 36% of trips required overnight air travel, with this potentially putting athletes at risk of impaired sleep [6, 10, 11]. Impairments in sleep may then have further implications for wellbeing and performance [5, 6, 10, 22], highlighting the need for appropriate strategies to monitor and promote sleep during travel [10, 11, 13]. Related to the overnight nature of travel, 33% of flights arrived in the first half of the day (24:00–12:00), and thus are likely to involve longer durations between full sleep periods which may have additional consequences for sleep and adaptation [23]. For such trips, daytime naps may be useful where athletes were unable to obtain sufficient sleep during travel [24]. Currently, no studies have reported jet lag, travel fatigue or other perceptual responses of national team footballers across varying travel demands. Although no specific jet lag or travel fatigue measures were available, based on the observations of previous research and the extent of travel observed in this study, it is likely that a considerable volume of national team travel may induce circadian misalignment, jet lag or sleep disruption. Hence, given the short-turnaround between club and national team fixtures, strategies to alleviate these consequences are recommended i.e. sleep hygiene, naps and awareness of travel schedules. Such strategies may be important in maximising the availability of players to train and prepare for both national team and club competition [24].

Understanding locational differences in travel demands of players travelling into national team commitments will enable staff to better cater to player-specific needs. Despite similarities in time zone difference for European- and Australian-based players, travelling from Europe required more eastward trips. Although not measured here, eastward trips are reported to induce more prolonged symptoms of jet lag [4, 20, 25] and may warrant earlier arrivals for European players or greater focus on interventions to hasten circadian adaptation. Asian-based players experienced significantly smaller time zone changes and thus the risk of jet lag when travelling into camp is less than that for European- or Australian-based players. Interestingly, players who were based at clubs in Australia had the greatest travel durations. Such a finding likely reflects the unique situation of the Australian national team in which the country is geographically based in Oceania but competes under the Asian Confederation and thus often compete in Asia. While time zone changes may still be a concern for Australian-based players, the greater concern may result from travel fatigue due to longer travel durations and potential implications of long-duration flights [9, 19, 21]. However, as longhaul daytime travel ≤ 10 h has not been observed to affect performance and wellbeing [12], similar travel fatigue symptoms theoretically may persist in all groups given overnight travel requirements were similar [5, 6, 10]. The similarities in overnight travel amongst all players suggests interventions to reduce travel induced sleep loss should be of focus for national team practitioners for player arrival into camp. Accordingly, a need for attention on circadian re-entrainment exists for European-based players, while sleep-promoting interventions during and after travel are required for all players arriving for national team duties.

Given the prevalence of fixture congestion in elite football [1], returning players to clubs from national teams requires effective communication between national and club team staff to enhance player recovery and selection availability. During return travel, Australian-based players had the worst travel schedules, with more eastward trips, longer travel durations and more trips requiring overnight travel. These travel schedules may place the athletes at greater likelihood of jet lag due to the longer lasting effects following eastward travel [4, 26], while longer travel durations and overnight flights have previously been observed to reduce sleep and increase fatigue [5, 11]. Therefore, additional focus on hastening time zone adaptations in players returning to Australian clubs is suggested, while attempts to reduce sleep deficits from overnight travel are also recommended. Earlier arrivals have previously been observed to contribute to greater symptoms of jet lag due to longer durations between full sleep periods [23]; and should also be considered in Australian-based players who on average arrived significantly earlier than European-based players. While greater attention is required for Australian-based players, the average time zone differences of 3.5 ± 3.2 h may still be enough to induce jet lag in European-based players [8, 9, 19]. Given these players largely travelled westward on return to clubs, it is speculative whether these symptoms may potentially alleviate quicker than eastward travelling players [4, 20, 26].

Despite the novelty of these results, several limitations should be considered when interpreting these findings. Importantly, as this study did not obtain any measures of wellness, performance or sleep from players, any suggested effects of travel are based on previous research. Additionally, the travel demands represent a case study of one national team undertaking tournaments at that point of the time. Furthermore, while a broad date range was used there is likely a bias in the findings based on the location of tournaments. Different travel demands are therefore likely to be observed between other national teams and time frames. Lastly, while a majority of international travel is performed via aircraft, this study does not consider additional modes of transport i.e. road or railway travel and its potential effects on players, nor does it account for travel to and from the airport [8].

## CONCLUSIONS

Overall, this study provides a detailed case-study of the type and extent of travel involved in a national football team, while demonstrating that these demands are likely to differ based on a player’s club location. Travel for national team duties are diverse, and there remains many schedules that require planning to maximise performance and wellbeing. A single squad-wise approach to travel scheduling may not be appropriate as the nature of travel differs significantly between player’s club locations. For the Australian national team, travel into camp is likely most demanding for European-based players, while Australian-based players may be more at risk of negative travel consequences following return travel. As such, it is important to consider the specific demands of players on an individual or at least regional basis.
